# A New Explanation for the Attitude-Behavior Inconsistency Based on the Contextualized Attitude

**DOI:** 10.3390/bs13030223

**Published:** 2023-03-03

**Authors:** Yuan Yuan, Rui Sun, Jiajia Zuo, Xue Chen

**Affiliations:** 1 School of Business Administration, Huaqiao University, Quanzhou 362000, China; 2 Oriental Business Management Research Center, Huaqiao University, Quanzhou 362000, China

**Keywords:** attitude-behavior inconsistency, contextualized attitude, retrospective attitude, illusion of privacy empowerment, event-related potentials

## Abstract

Inconsistency between attitude and behavior is a major obstacle to research on the predictive power of attitudes on behavior. To clarify the mechanism underlying such inconsistency, we combined event-related potential (ERP) and questionnaires to explore the relationship between contextualized attitudes and retrospective attitudes in the context of illusion of privacy empowerment (IPE). When the participants read the IPE events (including platform empowerment intention, technique, result, etc.) on slides, we measured retrospective attitudes with questionnaires and recorded contextualized attitudes with ERPs. We found that individuals’ retrospective attitudes were different from contextualized attitudes: retrospective attitudes were mainly affected by the individual’s analytic system, while contextualized attitudes were mainly affected by the direct stimulus-response (i.e., heuristic system). Therefore, retrospective attitudes may not accurately reflect individual cognition in the immediate context, and inconsistency between attitudes and behavior may be caused by the mismatch between retrospective attitudes and immediate behavior. Our findings provide a more reasonable account of the relationship between attitudes and behavior.

## 1. Introduction

Attitudes and behaviors have long been a focus of psychological research [1,2,3], with researchers primarily investigating the relationship between attitudes and behaviors to reveal the predictive power of attitudes on behavior [4,5]. However, numerous studies have demonstrated that individuals often express their attitudes but do not act in accordance with those attitudes [6]. For instance, some individuals may have negative views of smoking but still engage in smoking behaviors, some may state their intention to lose weight but eat high-calorie food, and some users may be concerned about privacy but are willing to sacrifice it for convenience.

The existing explanations for attitude–behavior inconsistency mainly focus on exploring the characteristics of attitude. First, based on the theory of attitude strength, it is argued that when attitude strength is strong, the attitude is clear and can help individuals resist persuasive messages and maintain a high degree of behavioral consistency. When attitude strength is weak, the attitude is vague, and therefore no corresponding behavior will be produced [7,8,9]. Second, based on the theory of planned behavior, it is argued that subjective norm and perceived behavioral control will both affect the predictive role of attitude on behavior [10,11,12]. Third, based on the system view of attitude, it is argued that attitude is overall. When one component of an individual’s attitude (i.e., affective, cognitive, or behavioral) is inconsistent with the overall evaluation or other components, attitude ambivalence exists, and attitude is difficult to guide behavior [13,14,15,16].

Although these studies have explained the inconsistency between attitude and behavior from multiple perspectives, there are still some problems due to the limitations of research perspectives and methods. First, the importance of specific contexts has been ignored, and the relationship between attitude and behavior is only discussed in the background of psychological systems. Second, the existing studies mostly adopt questionnaire measurement methods, but the questionnaire results are only hypothetical responses to hypothetical situations, which are overall evaluations after individuals’ retrospective, processing and rational analysis, subject to recall and the influence of individual subjective factors [17]. It is difficult to scientifically and accurately reflect the cognitive process in a real situation.

The illusion of privacy empowerment (IPE) means that platforms grant users invalid privacy control rights and data sovereignty, giving them the illusion of autonomous choice. For instance, even if they refuse to authorize their contacts, they may still be recommended related friends, or nearby information even if they refuse to authorize their location [18]. As IPE emerges, users claim to be concerned about it, yet they still accept the recommended information with “manipulative” attributes, which is a new attitude–behavior inconsistency phenomenon in the digital era.

Event-related potential (ERP) technology, which has a millisecond-level high temporal resolution, can better simulate immediate privacy decision-making scenarios. In addition, this technology is viewed as a “magnifying glass” for observing psychological processes without directly asking users about their thoughts, memories, evaluations, or decision strategies [19], which can precisely locate individual cognitive processes without being easily influenced by individual subjectivity. Different from questionnaire measurement, neuroscience experiments provide different research questions that can be solved. The results of questionnaire measurement are based on the individual’s comprehensive analysis and are mostly general and holistic perceptions and evaluations [20]. ERP technology is used to measure behaviors and cognitive processes directly generated by stimulus-response, which is based on heuristic processing and is the embodiment of instantaneous and emotional attitudes [19].

Based on this, this study takes IPE as its research context and employs a combination of questionnaires and ERP to measure the willingness to accept recommendation advertisements as a reflection of retrospective attitudes and cognitive resources such as attention to reflect contextualized attitudes. The study aims to explore the relationship between contextualized and retrospective attitudes to explain the inconsistency between individual attitudes and behaviors.

## 2. Literature Review and Research Hypothesis

### 2.1. Attitude–Behavior Inconsistency and the IPE

The attitude–behavior inconsistency has received widespread attention from scholars once it was proposed. Scholars have attempted to identify the causes of inconsistency, explore the factors restricting the relationship between attitude and behavior, and clarify the role of attitude in predicting behavior [4,5,6].

The research on attitude–behavior inconsistency mainly focuses on exploring the characteristics of attitude itself. First, the Dual Attitudes Model states that people can simultaneously hold two different evaluations for the same attitude object, one being an automatic, implicit attitude, and the other being an aware, explicit attitude [21]. Studies have demonstrated that behavior is the result of the combined effect of implicit and explicit attitudes. When the motivation level of the explicit attitude is low, individuals will take actions that are inconsistent with it [22,23,24]. Secondly, the Tripartite Model of Attitude Structure viewpoint suggests that attitude consists of cognition, emotion, and intention, and the consistency of evaluation among these components should be taken into consideration when examining the relationship between attitude and behavior [1,13,25]. Thirdly, the concept of attitude strength proposes that strong attitudes will affect the selection and processing of information, impede attitude changes, and influence the duration of attitude, thus exhibiting better behavior prediction ability [26,27,28].

However, some studies have also shown that individual behavior is often a product of the interaction between attitudes and their perceived context, arguing that even a specific attitude is not guaranteed to determine a person’s behavior in a given situation. It has led to the concept of contextualized attitudes, which refers to forming immediate attitudes based on changes in context [29,30,31]. Although the critical role of context in attitude–behavior inconsistency has been recognized, the limitations of research methods have made it difficult to further explore this topic.

The essence of the IPE is to manipulate users covertly by giving them the illusion of privacy control and autonomy of choice, then depriving them of their rights and interests. Individuals often show inconsistent attitudes and behaviors in the face of IPE. In IPE situations, users’ actual decision-making behavior is specific and immediate [32], and is only influenced by contextualized attitudes induced by contextual cues. Therefore, this study takes IPE as the research context to investigate the relationship between the contextualized attitude and retrospective attitude induced by different regulatory focus and social distance. By measuring the changes of contextualized attitude and retrospective attitude, this study aims to provide a new explanation for the individual’s attitude–behavior inconsistency phenomenon.

### 2.2. Regulatory Focus and Social Distance

Regulatory focus and social distance play a key role in affecting individuals’ privacy decisions. Therefore, scholars often use them in studies on privacy disclosure, information risk, and privacy-protective behavior [33,34,35].

Regulatory focus is an individual’s tendency to change or control their thoughts and reactions in a specific way and tendency to achieve a goal. It is divided into promotion focus and prevention focus [36,37,38]. Individuals with promotion focus are more likely to rely on emotional factors, focus on the potential benefits, and often ignore potential risks [39,40,41]. In contrast, those with prevention focus are more cognitively oriented, tend to avoid negative outcomes, and are more likely to search for relevant material and analyze potential risks rationally, even if they can benefit [42,43,44]. Therefore, it is hypothesized that promotion-focused individuals are more likely to change their attitudes toward privacy events when learning content related to privacy events because of the apparent hazards, while prevention-focused individuals will sort and judge the learned content, and their attitudes are less likely to change.

Based on this, this paper proposes the following hypothesis:

**H1.**  *The change in willingness to accept recommended information before and after learning of IPE events is more significant in individuals with promotion focus than in those with prevention focus.*

Social distance is a concept that refers to the perceived distance between an individual and other object in their environment. When making behavioral decisions, individuals frequently consider the proximity of interaction objects to themselves [45,46]. When the social distance is distal, individuals have fewer fair considerations and fewer emotional responses, which allows them to make rational decisions. When the social distance is proximal, individuals have higher expectations of social norms. When they are treated unfairly, triggering emotions such as surprise and frustration that usually lead individuals to make irrational decisions [47]. Compared to promotion-focused individuals, prevention-focused individuals who want to change their IPE attitudes require more redundant information to refine them. However, even in the case of proximal social distance, it is not easy for individuals to obtain enough information, making it difficult to have a large attitude change.

Based on this, this paper proposes the following hypotheses:

**H2.**  *In the promotion focus group, individuals’ willingness to accept recommendation information exhibits a greater change in the proximal social distance group than in the distal social distance group.*

**H3.**  *In the prevention focus group, social distance has no significant impact on the change of willingness to accept recommendation information.*

### 2.3. EEG Component Hypothesis

According to Cognitive Resource Theory, human cognitive resources are limited and individuals allocate certain cognitive resources to learning and evaluating each task [48,49]. When the individual is highly invested in the learning task, all available cognitive resources are occupied, making them less able to effectively inhibit task-irrelevant stimuli and more susceptible to interference from irrelevant stimuli. Conversely, individuals with a low level of engagement in the learning task have sufficient cognitive resources to suppress the interference of irrelevant stimuli [50]. Therefore, the present study employed the ERPs technique to scan and record subjects’ EEG signals using the single-stimulus experimental paradigm, replacing the non-target word sounds in the oddball paradigm with silent voice. The N1 and P2 component wave amplitudes reflect the degree of attentional distraction from irrelevant factors during the learning process, thereby laterally reflecting the input of cognitive resources in the individual learning process from another angle.

The N1 and P2 components have been reported to be related to attentional input and risk perception. As their attentional alertness increases, decision-makers are proposed to dedicate more attentional resources to stimuli. In addition, a higher level of attentional alertness is suggested to be associated with a greater N1 amplitude [51]. Meanwhile, the more attentional resources an individual puts into information processing, the higher the P2 amplitude is expected to be. It has also been claimed that negative stimuli may necessitate more attentional resources, leading to a higher P2 amplitude [52]. Moreover, when it comes to assessing the riskiness of safety sign warnings, research has shown that high-risk warnings tend to generate more P2 amplitude than low-risk warnings [53].

Both promotion and prevention focus individuals increased their attentional alertness in the face of the negative event of IPE, so there were no significant differences in N1 and P2 wave amplitudes between the promotion and prevention focus groups. Promotion-focused individuals are susceptible to simple information cues that do not occupy or occupy less cognitive resources [40]. Thus, the hazards embodied by social distance have no significant effect on them. On the other hand, prevention-focused individuals devote a large number of cognitive resources to identifying and evaluating the presented information [44], and proximal social distance events are more likely to cause them to perceive the threat and thus devote more attentional resources to them.

Based on this, the study proposes the following hypothesis:

**H4.**  *There is no significant difference in the N1 amplitude between the promotion focus group and the prevention focus group.*

**H5.**  *For promotion-focused individuals, there is no significant difference in the N1 amplitude between the distal distant group and proximal social distance group.*

**H6.**  *For prevention-focused individuals, the N1 amplitude in the distal social distance group is higher than that in the proximal social distance group.*

**H7.**  *There was no significant difference in the P2 amplitude of the promotion focus group and the P2 amplitude of the prevention focus group.*

**H8.**  *For promotion-focused individuals, there is no significant difference in the P2 amplitude between the distal distant group and proximal social distance group.*

**H9.**  *For prevention-focused individuals, the P2 amplitude of the distal social distance group was higher than the P2 amplitude of the proximal social distance group.*

**Figure 1:**
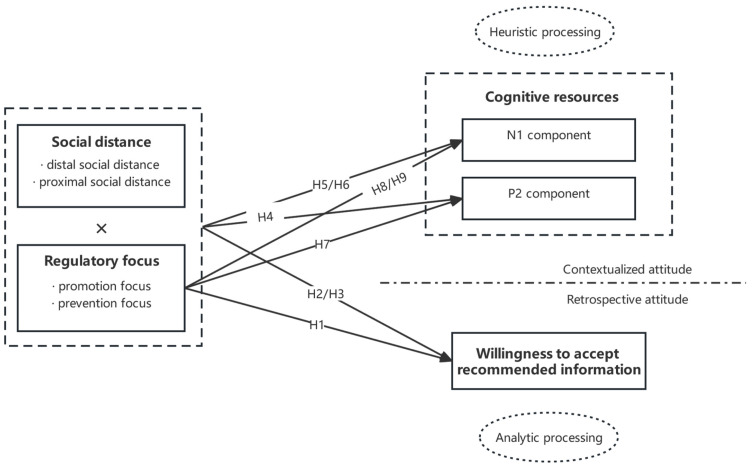
Research model.

The research model in this study is shown in Figure 1.

## 3. Materials and Methods

### 3.1. Participants

In this study, subjects willing to participate in the EEG experiment were randomly recruited through various social platforms, posters, and other publicity methods. Applicants were required to fill out a questionnaire covering personal information, contact information, regulatory focus items, and willingness to accept the recommendation information items. One hundred and sixty questionnaires were collected during the preliminary recruitment process. Among respondents, 88 were males and 72 were females, with an average age of 21 years old; 61.25% were undergraduate students, and 38.75% were graduate students. The regulatory focus questionnaire was adapted from the Regulatory Focus Questionnaire (Chinese Version) revised by Yao et al. (2010) [54], which is suitable for the Chinese context. It contained 10 items, including 6 promotion focus items and 4 prevention focus items. The score of regulatory focus is the score of the prevention focus questionnaire minus the score of the promotion focus questionnaire. The higher the score is, the more prevention-focused the individual tends to be, and the lower the score is, the more promotion-focused the individual tends to be. The scale of willingness to accept recommendation information is based on the willingness to accept recommendation advertisements. It includes three items: “I would be willing to accept information sent to me by this platform”, “I would be willing to click and browse information sent to me by this platform”, and “I would consider sharing information sent to me by this platform with others” [55]. Seven-point Likert scales were employed in both questionnaires, ranging from 1 (strongly disagree) to 7 (strongly agree).

G* Power3.1 was used to calculate the sample size required for research [56]. According to Cohen’s standard [57], F tests were used as the test family, and the parameters were set as follows: Repeated measures, within-between interaction, Effect size f=0.25, α err prob =0.05 $\mathrm{Power}\;\left(1-\beta \;\mathrm{err}\;\mathrm{prob}\right)=0.8$, Number of groups =4, Number of measurements=3, Corr among rep measures =0.5, Nonsphericity correction ε =1. The total sample size was 40. So, grouped by the level of regulatory focus scores, 20 participants with promotion focus and 20 participants with prevention focus were selected.

Then the participants of the EEG experiment were selected according to the questionnaire results. The specific selection method was as follows: We first ranked the regulatory focus scores of 160 respondents from highest to lowest, selected the 20 respondents with the highest scores as the participants in prevention focus group, and selected the 20 respondents with the lowest scores as the participants in promotion focus group. Among them, the score for promotion focus group was between −1.08~0.83 points, M=−0.17, SD=0.58, and the score for prevention focus group was between 1.25~2.41 points, M=1.59, SD=0.36. Among the participants, there were 17 males and 23 females, with an average age of 23. Undergraduate students accounted for 57.5%, and graduate students accounted for 42.5%.

The promotion focus group and the prevention focus group were randomly divided into two groups: the distal social distance group and the proximal social distance group. There was no significant difference in regulatory focus scores of the distal/proximal social distance participants in promotion focus group, $t_{\left(18\right)}=-0.146,\;p>0.05$. There was no significant difference in the regulatory focus scores of the distal/proximal social distance participants in prevention focus group, $t_{\left(18\right)}=-0.312,\;p>0.05$. There was no significant difference in age among the four groups, $F_{\left(3,27\right)}=0.727,\;p>0.05$. All subjects were right-handed, had no history of mental illness, and had normal or corrected-to-normal vision.

### 3.2. Procedure

We randomly selected 40 events (intentions, methods, and consequences) with distal social distance and 40 with proximal social distance. Subsequently, 50 participants were randomly chosen to read the materials and respond to the question of “To what extent do you think this event is an IPE event?” on a seven-point Likert scale (ranging from 1 (strongly disagree) to 7 (strongly agree)). The 20 materials with the highest scores were selected for the formal experiment.

#### Formal Experiment

In the single-stimulus experimental paradigm, this experiment used a silent speech to replace the non-target word sounds in the oddball paradigm, recorded the participants’ attention and cognitive resource input in the reading process, and explored the cognitive neural mechanisms in the real-time situations from the cognitive and attentional perspectives. The experiment adopted an inter-group design based on regulatory focus (promotion/prevention) * social distance (distal/proximal).

Participants were asked to watch a 10-min slide with an electroencephalogram (EEG) cap on. The content of the slide is typical IPE materials selected by the subject personnel through a strict manipulation test. Participants in the proximal social distance group read the domestic IPE events, while participants in the distal social distance group read foreign IPE events. The learning time, environment, content, and cognitive ability of the participants were all controlled at the same level.

Participants were asked to watch the content in the slide carefully. They were asked to fill out a questionnaire about their willingness to accept recommended information at the end of the experiment. Moreover, they were asked to recall the content of the IPE event in the slide and record it on paper in the form of keywords to measure their perception of the exogenous learning content.

### 3.3. Data Collection and Analysis

An American Neuroscan EEG recording and analysis system with a 64-channel Ag/AgCl electroencephalogram cap (according to the extended international 10–20 system) was employed in this study. The filter bandpass was set to 0.01–100 Hz, the sampling frequency to 100 Hz, and the resistance was maintained below 5 kΩ throughout the experiment. Both VEO and HOE were recorded.

Prior to the experiment, the participants were instructed to sit comfortably in a quiet, softly lit room with sound insulation. The distance between the participant’s eyes and the computer screen was approximately 1 m, and the horizontal and vertical viewing angles were set to less than 5°. During participants’ reading materials, a 1000 Hz (100 ms duration, 10 ms rise/fall time, and 60 dB SPL) audio stimulus was delivered binaurally at a distance of 60 cm from each ear, according to the single-stimulus experimental paradigm.

## 4. Results

### 4.1. Behavioral Data

*t*-test was carried out to compare the 40 participants’ willingness to accept the recommendation information before and after the experiment. There was no significant difference for the pre-experimental willingness to accept information between the promotion focus group $\left(M=4.06,\;\mathrm{SD}=0.87\right)$ and the prevention focus group $\left(M=3.86,\;\mathrm{SD}=0.73\right)$, $t_{\left(38\right)}=0.82$, p=0.420>0.05, see Table 1. The willingness to accept recommendation information in the promotion focus group ($t_{\left(19\right)}=6.15$, p<0.001) and the prevention focus group ($t_{\left(19\right)}=5.03$, p<0.001) were significantly different before and after the experiment, see Table 2. The change of willingness to accept recommendation information in the promotion focus group $\left(M=-1.17,\;\mathrm{SD}=0.85\right)$ was significantly higher than that in the prevention focus group (M=−0.53, SD=0.47), $t_{\left(38\right)}=-2.95$, p=0.005<0.05, see Table 3.

The willingness to accept the recommended information before and after the experiment of the prevention focus group and the promotion focus group were tested with T-test. There was no significant difference in the pre-experimental willingness to accept information between the distal/proximal social distance group of promotion focus ($t_{\left(18\right)}=0.78$, p=0.448>0.05) and prevention focus ($t_{\left(18\right)}=-0.04$, p=0.970>0.05), see Table 1. In the promotion focus group, the willingness to accept information before and after the distal ($t_{\left(9\right)}=4.21$, p=0.002<0.01)/proximal ($t_{\left(9\right)}=5.39$, p<0.001) social distance experiment was significantly different, see Table 2. The change in the willingness to accept information from the proximal social distance group (M=−1.57,SD=0.92) was significantly higher than that of the distal social distance group (M=−0.78,SD=0.58),$\;t_{\left(18\right)}=-2.29$, p=0.034<0.05, see Table 3. In the prevention focus group, the willingness to accept information before and after the distal ($t_{\left(9\right)}=3.80$, p=0.004<0.01)/proximal ($t_{\left(9\right)}=3.26$, p=0.01<0.05) social distance experiments were significantly different, see Table 2. However, there was no significant difference in the change range of willingness to accept information between distal social distance (M=−0.41, SD=0.43) and proximal (M=−0.65, SD=0.51) social distance,$t_{\left(18\right)}=1.11$, p=0.283>0.05, see Table 3.

### 4.2. Event-Related Potential Data

#### 4.2.1. N1 Component

According to the overall superimposed butterfly diagram, the N1 component time window was selected for 120–200 ms, and three electrodes (PO3, POZ, PO4) were selected as representative sites. We performed a 2 (regulatory focus: promotion focus, prevention focus) × 3 (electrode site: PO3, POZ, PO4) within-subjects repeated measure ANOVA on N1 amplitudes. The result implies that the Mauchly sphericity test is nonconforming, p=0.013<0.050; hence, we used multivariate tests. The multivariate tests show that main effect of electrode site was significant ($F_{\left(2,37\right)}=6.37$, p=0.004<0.05), and the interaction between electrode site and regulatory focus was not significant, $F_{\left(2,37\right)}=0.21$, p=0.811>0.050. See Table 4.

**Figure 2:**
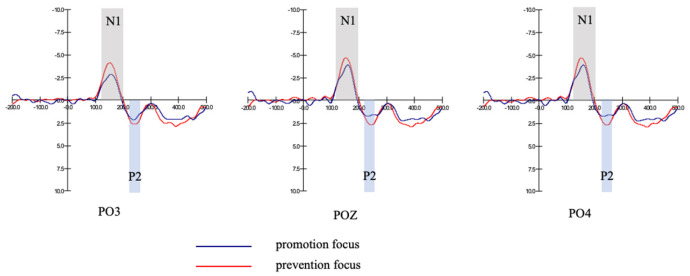
N1, P2 amplitudes of different regulatory focus types.

Main effect of regulatory focus was not significant ($F_{\left(1,38\right)}=2.72$, p=0.107>0.05), see Table 5. There was no significant difference in N1 amplitude between different types of regulatory focus. Figure 2 gives the overall picture of grand-averaged ERP waveforms for promotion focus and prevention focus at different sites (PO3, POZ, PO4).

Both groups of participants for promotion focus and prevention focus were then further divided into distal social distance and proximal social distance, and performed a 2 (social distance: distal/proximal) × 3 (electrode site: PO3, POZ, PO4) within-subjects repeated measure ANOVA. For the promotion focus group, the result implies that Mauchly sphericity test is nonconforming, p=0.032<0.050; hence, we used multivariate tests. The multivariate tests show that the main effect of electrode site was significant ($F_{\left(2,17\right)}=4.40$, p=0.029<0.05), and the interaction between electrode site and social distance was not significant, $F_{\left(2,17\right)}=2.33$, p=0.128>0.050. See Table 6.

**Figure 3:**
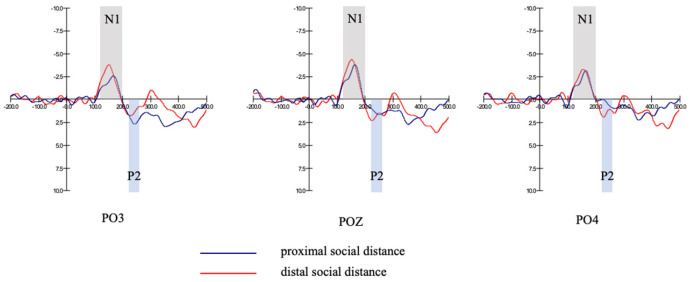
N1, P2 amplitudes of different social distance groups (promotion focus group).

The main effect of social distance was not significant ($F_{\left(1,38\right)}=0.56$, p=0.465>0.05), see Table 7. There was no significant difference in N1 amplitude between the distal and proximal social distance groups. Figure 3 shows grand-averaged ERP waveforms evoked by distal social distance and proximal social distance (in the promotion focus group) at PO3, POZ, PO4.

For the prevention focus group, the result implies that the Mauchly sphericity test is conforming, p=0.113>0.050; hence, we used Within-Subjects Effects. The test of Within-Subjects Effects shows that the main effect of electrode site was not significant, $F_{\left(2,36\right)}=2.07$, p=0.14>0.05. The interaction between electrode site and social distance was significant $F_{\left(2,36\right)}=4.04$, p=0.026<0.05. See Table 8.

**Figure 4:**
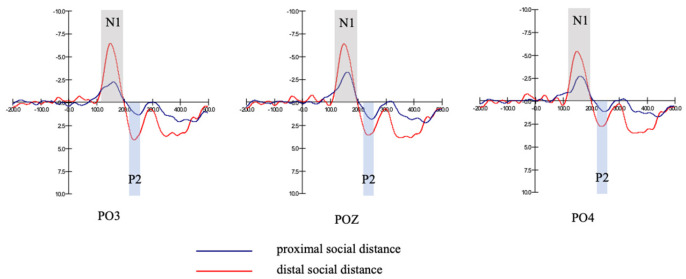
N1, P2 amplitudes of different social distance groups (prevention focus group).

From the estimated marginal means, the amplitude of the distal social distance group (M=−6.99, SD=0.65) was significantly higher than that of the proximal social distance group (M=−3.26, SD=0.65), $F_{\left(1,18\right)}=16.69$, p=0.001<0.01. Figure 4 shows grand-averaged ERP waveforms evoked by distal social distance and proximal social distance (in the prevention focus group) at PO3, POZ, PO4 (Table 9).

#### 4.2.2. P2 Component

According to the overall superimposed butterfly diagram, the P2 component time window was set as 220–260 ms, three electrodes (PO3, POZ, PO4) were selected as representative sites. We performed a 2 (regulatory focus: promotion focus, prevention focus) × 3 (electrode site: PO3, POZ, PO4) within-subjects repeated measure ANOVA on P2 amplitudes. The result implies that the Mauchly sphericity test is nonconforming, p=0.000<0.050; hence, we used multivariate tests. The multivariate tests show that the main effect of electrode site was significant ($F_{\left(2,37\right)}=4.10$, p=0.025<0.05), the interaction between electrode site and regulatory focus was no significant, $F_{\left(2,37\right)}=2.66$, p=0.083>0.050. See Table 10.

The main effect of regulatory focus was not significant ($F_{\left(1,38\right)}=0.00$, p=0.986>0.05), see Table 11. There was no significant difference in N1 amplitude between different types of regulatory focus. Figure 2 gives the overall picture of grand-averaged ERP waveforms for promotion focus and prevention focus at different sites (PO3, POZ, PO4).

Next, with both groups of subjects for promotion focus and prevention focus divided by the social distance factor (namely distal social distance and proximal social distance), we performed a 2 (social distance: distal/proximal) × 3 (electrode site: PO3, POZ, PO4) within-subjects repeated measure ANOVA. For the promotion focus group, the result implies that the Mauchly sphericity test is nonconforming, p=0.000<0.050; hence, we used multivariate tests. The multivariate tests show that main effect of electrode site was not significant ($F_{\left(2,17\right)}=0.33$, p=0.727<0.05), and the interaction between electrode site and social distance was significant, $F_{\left(2,17\right)}=4.22$, p=0.032<0.050. See Table 12.

Main effect of social distance was not significant ($F_{\left(1,18\right)}=0.32$, p=0.577>0.05), see Table 13. There was no significant difference in P2 amplitude between the distal and proximal social distance groups. Figure 3 shows grand-averaged ERP waveforms evoked by distal social distance and proximal social distance (in the promotion focus group) at PO3, POZ, PO4.

For the prevention focus group, the result implies that the Mauchly sphericity test is conforming, p=0.246>0.050; hence, we used Within-Subjects Effects. The test of Within-Subjects Effects shows that the main effect of electrode site was not significant, $F_{\left(2,36\right)}=12.05$, p=0.000<0.001. The interaction between electrode site and social distance was significant $F_{\left(2,36\right)}=1.57$, p=0.034<0.05. See Table 14.

From the estimated marginal means, the amplitude of the distal social distance group (M=−6.99, SD=0.65) was significantly higher than that of the proximal social distance group (M=−3.26, SD=0.65), $F_{\left(1,18\right)}=4.45$, p=0.049<0.05. Figure 4 shows grand-averaged ERP waveforms evoked by distal social distance and proximal social distance (in the prevention focus group) at PO3, POZ, PO4 (Table 15).

## 5. Discussion

The effects of regulatory focus on individual attitudes. The questionnaire data showed that the willingness to accept recommended information before and after reading IPE events was significantly greater for promotion-focused individuals than for prevention-focused individuals. Previous research has shown that prevention-focused individuals can analyze information more deeply and integrate new information with existing ones when they encounter different cognition from themselves [42,43]. In contrast, promotion-focused individuals often analyze events based on emotions and superficial information, which makes them more likely to change their attitudes [39,40]. Therefore, prevention-focused individuals have a greater change in willingness to accept recommended information compared to promotion-focused individuals. However, when it comes to the immediate context, there was no significant difference in the attention resources devoted to the privacy authorization event by individuals with different regulatory focus (as indicated by the N1 and P2 amplitudes). This implies that individuals with different regulatory focus have similar resistance to external stimuli when reading the IPE events, and the cognitive resources and attention devoted are not significantly different. The inherent information analysis mode of different personality traits does not directly affect the immediate attention input and risk perception.

The effect of the interaction between regulatory focus and social distance on individual attitudes. The questionnaire data showed that the change in the willingness to accept recommended information was significantly greater for individuals with promotion focus who were in the proximal social distance group than for those who were in the distal social distance group. There was no significant effect of social distance on the change in willingness to accept recommended information in prevention focus group. The study demonstrated that individuals with promotion focus tended to rely on intuition or direct cues from the surrounding environment to make decisions [41]. In contrast, individuals with promotion focus had higher cognitive demands, refined external knowledge more accurately, and recognized the nature of the information [58,59]. Thus, for the promotion focus group, when reading the domestic IPE events, they had a stronger sense of immersion and were more likely to recognize the essence of manipulation, so they were more likely to change their original willingness to accept recommended information, thus leading to a more significant change.

This indicates that although regulatory focus does not directly affect cognition in immediate contexts, it can affect individuals’ recognition of direct cues and induce inconsistent heuristic processing. Promotion-focused individuals are susceptible to simple information cues, whereas prevention-focused individuals have more cognitive demands [39,58]. Thus, promotion-focused individuals do not significantly differ in the attentional resources they devote to IPE events with different social distances and their perceived risk. In contrast, prevention-focused individuals are more sensitive to social distance in immediate contexts, devote more cognitive resources to events with proximal social distance (highly self-relevant), and increase their risk perception.

## 6. Conclusions

This study adopted ERPs experiments to explore the relationship between contextualized attitudes and retrospective attitudes, aiming to explain the attitude–behavior inconsistency. The participants were asked to read IPE-related events (including platform empowerment intentions, methods, results, etc.). This study recorded and analyzed the willingness to accept recommended information before and after the experiment, as well as the N1 and P2 components related to attention investment.

The conclusions of this study are as follows. The questionnaire data showed that the willingness of participants to accept recommendation information changed more significantly after learning about the IPE events for promotion-focused individuals than for prevention-focused individuals. For promotion-focused individuals, the difference in the change of willingness to accept recommendation information after reading proximal social distance IPE events is greater than that after reading distal social distance IPE events. For prevention-focused individuals, social distance has no significant impact on the change of willingness to accept recommendation information. However, the ERPs results show that the changes in attitudes seem unrelated to users’ cognition in the immediate context, and the attitude measured by the questionnaire can barely reflect the individual cognition in the immediate context. The questionnaire results show that retrospective attitudes are mainly affected by the individual analytical system, while the EEG results show that immediate contextualized cognition is mainly affected by the direct stimulus-response (i.e., the heuristic system) and the inconsistency between individual attitude and behavior is probably only the difference between retrospective attitude and immediate behavior. Specifically, individual attitudes are updated and iterated in real-time [60]. Nevertheless, most previous studies were restrained by the inherent limitations in their methodology and focused on the relationship between attitude and behavior from the retrospective attitude perspective [17], which is a stable attitude formed by analyzing the recently obtained information and behaviors [17,20]. In most cases, it is like, “I do not know until you ask me”, so it is difficult to match with the immediate behavior.

This study has certain theoretical implications. First, by introducing the contextualized attitude, the intrinsic cognitive process of individual behavior is clarified, and the differences between the contextualized attitude and the retrospective attitude are discussed, which effectively explains why the past attitude is difficult to correspond to users’ actual behavior. Previous studies mostly focus on the impact of overall evaluation on individual behavior [20], but the research shows that individual behavior is deeply affected by context and individual emotions [29,30,31], and retrospective attitudes are not enough to explain individual behavior, and contextualized attitudes are important antecedents influencing privacy behavior. Second, the combination of EEG experiments and post-event questionnaires is used to make up for the limitations of questionnaire methods, such as insufficient explanation and vague real-life contexts. It can be said that the results of the questionnaire are mostly hypothetical responses to hypothetical contexts due to a retrospective design. They are easily affected by memory and cognitive bias [17,20], making it difficult to predict individual behavior accurately. In contrast, the results of EEG experiments reflect the direct “stimulus-response” in specific decision-making contexts. Combining the two research methods not only enhances the scientific and logical nature of the experimental design, but also improves the credibility of the research conclusions. At the same time, it can also provide a methodological reference for subsequent related research.

Clarifying the explanatory mechanism of attitude–behavior inconsistency can reasonably explain the relationship between attitude and behavior, effectively minimize the factors affecting attitude–behavior inconsistency, and thus improve the consistency between attitude and behavior. It can also improve the predictive power of attitude towards behavior in areas such as consumption, management, crime, health, etc., such as guiding enterprises to promote consumer psychology into consumer behavior, and guiding politicians to transform individual political inclinations into political choice behaviors.

Although this study has certain theoretical and practical significance, some things could be improved. First, all participants are college students, which may decrease the research results’ external validity. However, college students use the internet more frequently than other groups and thus experience more IPE events. Moreover, students come from all over the country, with different cultures, concepts, and backgrounds, thus making the research samples diverse, which can minimize sampling errors as much as possible. Hence, the research results still have certain effects. Second, this study only discussed the effect of attention on attitude change in the immediate context of encountering IPE events. Subsequent studies will explore the relationship between cognitive processes such as emotion and cognitive conflict and immediate decision-making in different contexts. In addition, as attitude change may be an iterative and accumulative process [60], subsequent research will analyze EEG signals to explain the process of individual attitude change with the relationship between alpha waves and reaction time.

## Figures and Tables

**Table 1:** Independent samples test (pre-experimental willingness to accept information).

		Levene’ Test for Equality of Variances		*t*-Test for Equality of Means						
		*F*	*Sig.*	*t*	df	*Sig.* (2-Tailed)	Mean Difference	Std. Error Differece	95% Confidence Interval of the Difference	
									Lower	Upper
promotion focus vs. prevention focus	Equal variances assumed	1.85	0.181	0.82	38	0.42	0.21	0.25	−0.31	0.72
	Equal variances not assumed			0.82	36.89	0.42	0.21	0.25	−0.31	0.72
distal social distance vs. proximal social distance (promotion focus)	Equal variances assumed	1.50	0.236	0.78	18	0.448	0.31	0.39	−0.52	1.13
	Equal variances not assumed			0.78	16.04	0.449	0.31	0.39	−0.53	1.14
distal social distance vs. proximal social distance (prevention focus)	Equal variances assumed	0.28	0.6	−0.04	18	0.97	−0.01	0.34	−0.72	0.69
	Equal variances not assumed			−0.04	16.77	0.97	−0.01	0.34	−0.72	0.70

**Table 2:** Paired samples test (the willingness to accept information before and after experiment).

		Paired Difference					*t*	*df*	*Sig.* (2-Tailed)
		Mean	Std. Deviation	Std. Error Mean	95% Confidence Interval of the Difference				
					Lower	Upper			
promotion focu	total	1.17	0.85	0.19	0.77	1.57	6.15	19	0.000
	distal social distance	0.78	0.58	0.18	0.36	1.20	4.21	9	0.002
	proximal social distance	1.57	0.92	0.29	0.91	2.23	5.39	9	0.000
promotion focu	total	0.53	0.47	0.11	0.31	0.75	5.03	19	0.000
	distal social distance	0.63	0.52	0.17	0.25	1.00	3.80	9	0.004
	proximal social distance	0.43	0.42	0.13	0.13	0.73	3.26	9	0.010

**Table 3:** Independent samples test (change range of willingness to accept information).

		Levene’ Test for Equality of Variances		*t*-Test for Equality of Means						
		*F*	*Sig.*	*t*	*df*	*Sig.* (2-Tailed)	Mean Difference	Std. Error Differece	95% Confidence Interval of the Difference	
									Lower	Upper
promotion focus vs. prevention focus	Equal variances assumed	7.10	0.01	−2.95	38	0.005	−0.64	0.22	−1.08	−0.20
	Equal variances not assumed			−2.95	29.63	0.006	−0.64	0.22	−1.09	−0.20
distal social distance vs. proximal social distance (promotion focus)	Equal variances assumed	2.24	0.151	−2.29	18.00	0.034	−0.79	0.34	−1.51	−0.06
	Equal variances not assumed			−2.29	15.25	0.037	−0.79	0.34	−1.52	−0.06
distal social distance vs. proximal social distance (prevention focus)	Equal variances assumed	0.14	0.712	1.11	18.00	0.283	0.23	0.21	−0.21	0.67
	Equal variances not assumed			1.11	17.50	0.283	0.23	0.21	−0.21	0.67

**Table 4:** Multivariate tests of N1 amplitude (regulatory focus).

		Value	*F*	Hypothesis *df*	Error *df*	*Sig.*
electrode site	Pillai’s Trace	0.26	6.37	2	37	0.004
	Wilks’ Lambda	0.74	6.37	2	37	0.004
	Hotelling’s Trace	0.34	6.37	2	37	0.004
	Roy’s Largest Root	0.34	6.37	2	37	0.004
electrode site * regulatory focus	Pillai’s Trace	0.01	0.21	2	37	0.811
	Wilks’ Lambda	0.99	0.21	2	37	0.811
	Hotelling’s Trace	0.01	0.21	2	37	0.811
	Roy’s Largest Root	0.01	.	2	37	0.811

**Table 5:** Tests of Between-Subjects Effects of N1 amplitude (regulatory focus).

	Type III Sum of Squares	*df*	Mean Square	*F*	*Sig.*
Intercept	2423.82	1	2423.82	140.07	0.000
regulatory focus	47.11	1	47.11	2.72	0.107
Error	657.57	38	17.31		

**Table 6:** Multivariate tests of N1 amplitude (promotion focus).

		Value	*F*	Hypothesis *df*	Error *df*	*Sig.*
electrode site	Pillai’s Trace	0.34	4.40	2	17	0.029
	Wilks’ Lambda	0.66	4.40	2	17	0.029
	Hotelling’s Trace	0.52	4.40	2	17	0.029
	Roy’s Largest Root	0.52	4.40	2	17	0.029
electrode site * social distance	Pillai’s Trace	0.22	2.33	2	17	0.128
	Wilks’ Lambda	0.79	2.33	2	17	0.128
	Hotelling’s Trace	0.27	2.33	2	17	0.128
	Roy’s Largest Root	0.27	2.33	2	17	0.128

**Table 7:** Tests of Between-Subjects Effects of N1 amplitude (promotion focus).

	Type III Sum of Squares	*df*	Mean Square	*F*	*Sig.*
Intercept	897.55	1	897.55	74.56	0.000
social distance	6.72	1	6.72	0.56	0.465
Error	216.70	18	12.04		

**Table 8:** Test of Within-Subjects Effects of N1 amplitude (prevention focus).

		Type III Sum of Squares	*df*	Mean Square	*F*	*Sig.*
electrode site	Sphericity Assumed	3.03	2	1.51	2.07	0.140
	Greenhouse-Geisser	3.03	1.63	1.86	2.07	0.151
	Huynh-Feldt	3.03	1.87	1.62	2.07	0.144
	Lower-bound	3.03	1.00	3.03	2.07	0.167
electrode site * social distance	Sphericity Assumed	5.90	2	2.95	4.04	0.026
	Greenhouse-Geisser	5.90	1.63	3.62	4.04	0.035
	Huynh-Feldt	5.90	1.87	3.15	4.04	0.029
	Lower-bound	5.90	1.00	5.90	4.04	0.060
Error (electrode site)	Sphericity Assumed	26.27	36	0.73		
	Greenhouse-Geisser	26.27	29.37	0.90		
	Huynh-Feldt	26.27	33.68	0.78		
	Lower-bound	26.27	18.00	1.46		

**Table 9:** Tests of Between-Subjects Effects of N1 amplitude (prevention focus).

	Type III Sum of Squares	*df*	Mean Square	*F*	*Sig.*
Intercept	1573.38	1	1573.38	125.70	0.000
social distance	208.85	1	208.85	16.69	0.001
Error	225.31	18	12.52		

**Table 10:** Multivariate tests of P2 amplitude (regulatory focus).

		Value	*F*	Hypothesis *df*	Error *df*	*Sig.*
electrode site	Pillai’s Trace	0.18	4.10	2	37	0.025
	Wilks’ Lambda	0.82	4.10	2	37	0.025
	Hotelling’s Trace	0.22	4.10	2	37	0.025
	Roy’s Largest Root	0.22	4.10	2	37	0.025
electrode site * regulatory focus	Pillai’s Trace	0.13	2.66	2	37	0.083
	Wilks’ Lambda	0.87	2.66	2	37	0.083
	Hotelling’s Trace	0.14	2.66	2	37	0.083
	Roy’s Largest Root	0.14	2.66	2	37	0.083

**Table 11:** Tests of Between-Subjects Effects of P2 amplitude (regulatory focus).

	Type III Sum of Squares	*df*	Mean Square	*F*	*Sig.*
Intercept	982.00	1	982.00	77.93	0.000
regulatory focus	0.00	1	0.00	0.00	0.986
Error	478.84	38	12.60		

**Table 12:** Multivariate tests of P2 amplitude (promotion focus).

		Value	*F*	Hypothesis *df*	Error *df*	*Sig.*
electrode site	Pillai’s Trace	0.04	0.33	2	17	0.727
	Wilks’ Lambda	0.96	0.33	2	17	0.727
	Hotelling’s Trace	0.04	0.33	2	17	0.727
	Roy’s Largest Root	0.04	0.33	2	17	0.727
electrode site * social distance	Pillai’s Trace	0.33	4.22	2	17	0.032
	Wilks’ Lambda	0.67	4.22	2	17	0.032
	Hotelling’s Trace	0.50	4.22	2	17	0.032
	Roy’s Largest Root	0.50	4.22	2	17	0.032

**Table 13:** Tests of Between-Subjects Effects of P2 amplitude (promotion focus).

	Type III Sum of Squares	*df*	Mean Square	*F*	*Sig.*
Intercept	492.91	1	492.91	45.84	0.000
social distance	3.47	1	3.47	0.32	0.577
Error	193.53	18	10.75		

**Table 14:** Test of Within-Subjects Effects of P2 amplitude (prevention focus).

		Type III Sum of Squares	*df*	Mean Square	*F*	*Sig.*
electrode site	Sphericity Assumed	10.15	2	5.08	12.05	0.000
	Greenhouse-Geisser	10.15	1.74	5.85	12.05	0.000
	Huynh-Feldt	10.15	2.00	5.08	12.05	0.000
	Lower-bound	10.15	1.00	10.15	12.05	0.003
electrode site * social distance	Sphericity Assumed	3.14	2	1.57	3.72	0.034
	Greenhouse-Geisser	3.14	1.74	1.81	3.72	0.041
	Huynh-Feldt	3.14	2.00	1.57	3.72	0.034
	Lower-bound	3.14	1.00	3.14	3.72	0.070
Error (electrode site)	Sphericity Assumed	15.17	36	0.42		
	Greenhouse-Geisser	15.17	31.25	0.49		
	Huynh-Feldt	15.17	36.00	0.42		
	Lower-bound	15.17	18.00	0.84		

**Table 15:** Tests of Between-Subjects Effects of P2 amplitude (prevention focus).

	Type III Sum of Squares	*df*	Mean Square	*F*	*Sig.*
Intercept	489.10	1	489.10	38.97	0.000
social distance	55.90	1	55.90	4.45	0.049
Error	225.94	18	12.55		

## Data Availability

The data presented in this study are available on request from the corresponding author.
